# Phenotypic plasticity in diaspore production of a amphi-basicarpic cold desert annual that produces polymorphic diaspores

**DOI:** 10.1038/s41598-020-67380-0

**Published:** 2020-07-07

**Authors:** Lu Gan, Juanjuan Lu, Jerry M. Baskin, Carol C. Baskin, Dunyan Tan

**Affiliations:** 1 College of Grassland and Environment Sciences, Xinjiang Agricultural University, Urümqi, China; 2 Department of Biology, University of Kentucky, Lexington, KY USA; 3 Department of Plant and Soil Sciences, University of Kentucky, Lexington, KY USA

**Keywords:** Plant ecology, Plant stress responses

## Abstract

Phenotypic plasticity has been studied in diaspore-dimorphic species, but no such study has been done on a diaspore-polymorphic species. Our aim was to determine the effects of abiotic and biotic factors on phenotypic plasticity of the diaspore-polymorphic cold desert annual *Ceratocarpus arenarius.* Plants produced from dispersal units near the soil surface (a, basicarps) and at the middle (c) and upper (f) parts of the plant canopy were subjected to different levels of soil moisture, nutrient supply and intramorph and intermorph densities. Different levels of these biotic and abiotic factors resulted in significant variation in total plant mass, diaspore mass, mass allocation to stem and reproductive organs and total number and proportion of morphs a, c and f on an individual. The effect of stress on number and mass of a dispersal unit morph varied by treatment, with dispersal unit f having the highest CV and dispersal unit a the lowest. The success of this diaspore polymorphic species in its rainfall-unpredictable environment likely is enhanced by plasticity in production of the different types of diaspores.

## Introduction

Fruit and seed heteromorphism is a phenomenon in which individual plants produce two or more kinds of diaspores that differ in size, mass, dispersal and dormancy^1^, and it may be a bet-hedging strategy that reduces the risk of failure under temporal environmental uncertainty^2^. Phenotypic plasticity, the capability of a genotype to produce different phenotypes in different environmental conditions, is a common phenomenon in plants^3–6^. It includes morphological^7–9^, physiological^10,11^ and ecological^12,13^changes in the phenotype and can occur in both diaspore monomorphic and diaspore heteromorphic species.

Plasticity in diaspore production has been studied in species with dimorphic aerial diaspores^1,14^. These studies have shown that stress can increase^15–20^, decrease^21–23^ or not change^24,25^ the proportion of the two diaspores. However, little is known about plasticity of diaspore production in species with three (trimorphic) diaspore morphs^26–28^ and even less about those with more than three (polymorphic) morphs^29–31^. Further, no such studies have been done on an amphi-basicarpic species that produces polymorphic diaspores.

**Figure 1:**
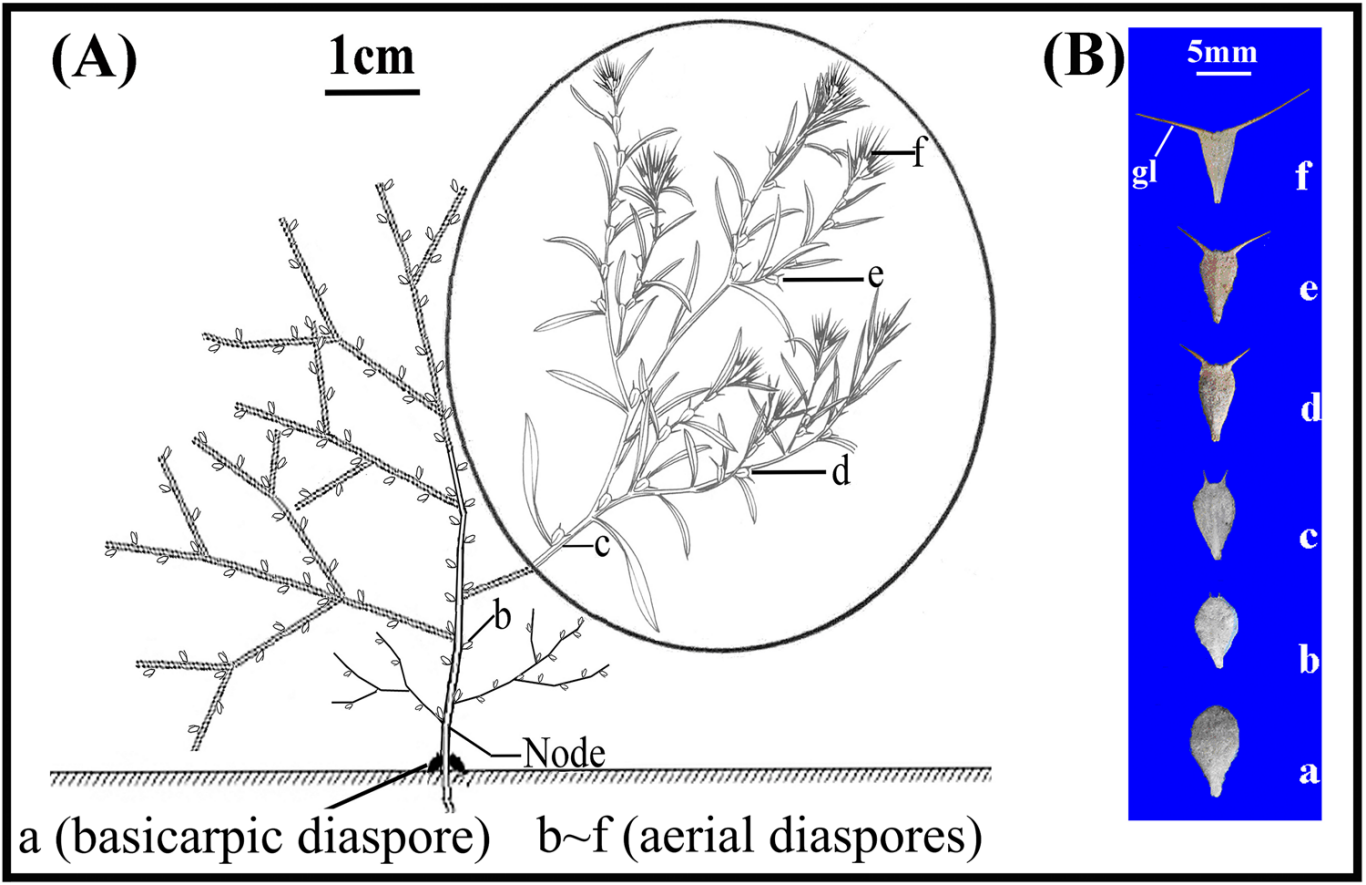
Position of basicarpic (a) and aerial (b–f) dispersal units of *Ceratocarpus arenarius* on an individual plant (**A**), and morphology of the six dispersal unit morphs of *Ceratocarpus arenarius* (**B**). The stem of this tumbleweed breaks at the node that is labeled. Circled material from (www.chinabaike.com). gl, glochid. (from Lu et al. 2015, with permission).

The summer annual polymorphic species *Ceratocarpus arenarius* L. (Amaranthaceae) is native to middle and central Asia, and in China it grows only in the cold deserts of northern Xinjiang Province of NW China^32^. Plants produce fruits (utricles) near the soil surface (basicarps) and a continuous series of morphologically distinct fruits from the lower to upper parts of the canopy. The (usually two) basicarps are designated as morph a and the canopy morphs b-f (from lower to upper part of canopy) (Fig. 1). Each fruit is covered by two bracteoles, and the fruit with these bracteoles is the dispersal and germination unit of the species. The number of aerial dispersal units per individual is 35–740, depending on plant size and habitat conditions (i.e. degree of stress)^33^. Thus, *C. arenarius* is an amphi-basicarpic species^1,34^ with polymorphic diaspores. Previous studies have shown that the polymorphic diaspores of this species differ in morphology, dispersal ability, intensity of dormancy^35^ and type of seed bank^33^.

Differences in morphology of basicarps (dispersal unit morph a) and aerial dispersal morphs (represented by c and f) of *C. arenarius* were correlated with differences in timing of germination and in capacity for dispersal. Relative dispersal ability was f > c > a, whereas relative intensity of dormancy was a > c > f^35^. Dispersal unit f has high dispersal-low dormancy (HDi-LDo), a “colonizer” type of strategy that can expand the range of the species to new sites where the seeds can germinate quickly. However, the chance that the seedlings will survive and become established is low, thus a high-risk strategy. On the other hand, dispersal unit a has low (or no) dispersal-high dormancy (LDi-HDo), a “maintainer” type of strategy. That is, the seeds remain in the parental habitat where conditions have proven to be suitable for germination, growth and reproduction of the species, thus a low-risk strategy. Dispersal unit c has dispersal and dormancy characteristics intermediate between the “colonizer” and “maintainer” types^35^.

Our field observations across the cold deserts of northern Xinjiang Province in NW China revealed that *C. arenarius* grows in a diversity of habitats (e.g. sand dunes, salty desert and desert steppe). Depending on the site, plants of this species vary considerably in size (from 5 to 35 cm in height) and in density within populations. In relatively low-nutrient habitats, plants may be dense but very small, but in other habitats with relatively deep/good soil plants are large and well spaced. These field observations lead us to speculate that the species exhibits phenotypic plasticity in size when grown in soils that differ in water availability and nutrients and when they encounter competition from neighbors^33^. Consequently, levels of soil moisture, nutrients and competition (plant density) potentially are environmental factors that contribute to variations in phenotypic responses of *C. arenarius* plants.

We tested the hypothesis that increasing stress causes a change in the ratio of dispersal unit morphs produced by plants. Specifically, we hypothesized that stress would cause a decrease in production of morphs b–f, which have low seed dormancy and high dispersal, and an increase in morph a, which has high seed dormancy and low dispersal. To test our hypothesis, we compared dry mass accumulation and mass allocation to vegetative and reproductive organs and dispersal unit production (fitness) in plants derived from dispersal unit morphs a, c and f. Plants from morphs a, c and f were grown under water and nutrient stress and in competition with plants derived from the same or different dispersal unit morph(s).

## Results

### Dry mass accumulation and allocation

**Figure 2:**
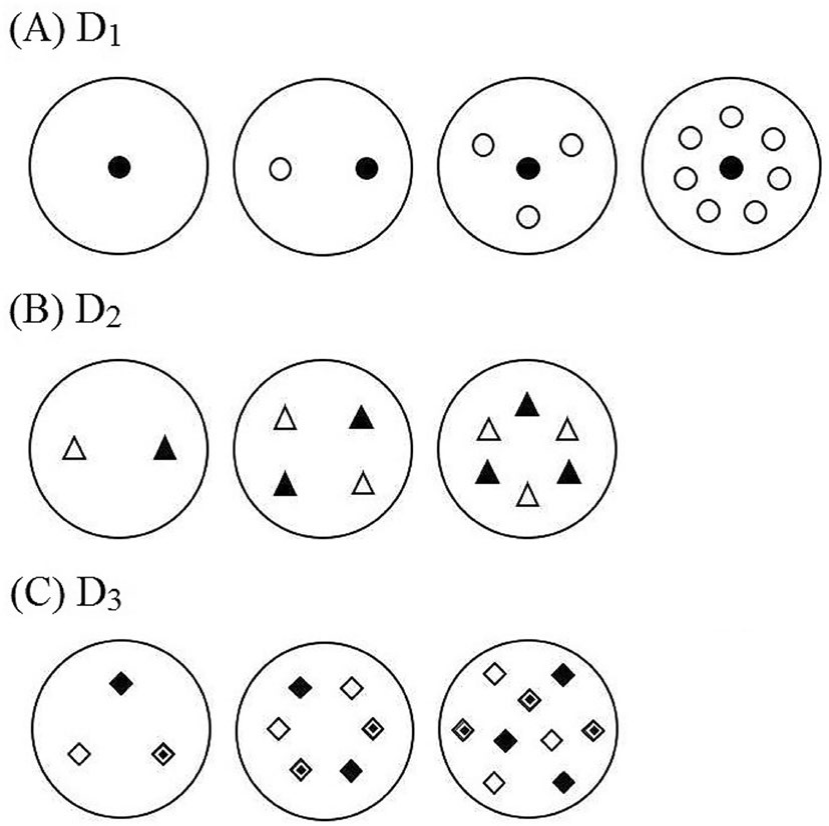
Distribution of *Ceratocarpus arenarius* plants from different dispersal unit morphs in pure (D_1_) (**A**) and mixed [D_2_ (**B**) and D_3_ (**C**)] densities. In (**A**), the target plant ( ) and its competitors from the same morph ( ); in (**B**), plants from a particular dispersal unit morph ( ) and the other morph ( ); and in (**C**), plants from dispersal units a ( ), c ( ) and f ( ).

**Figure 3:**
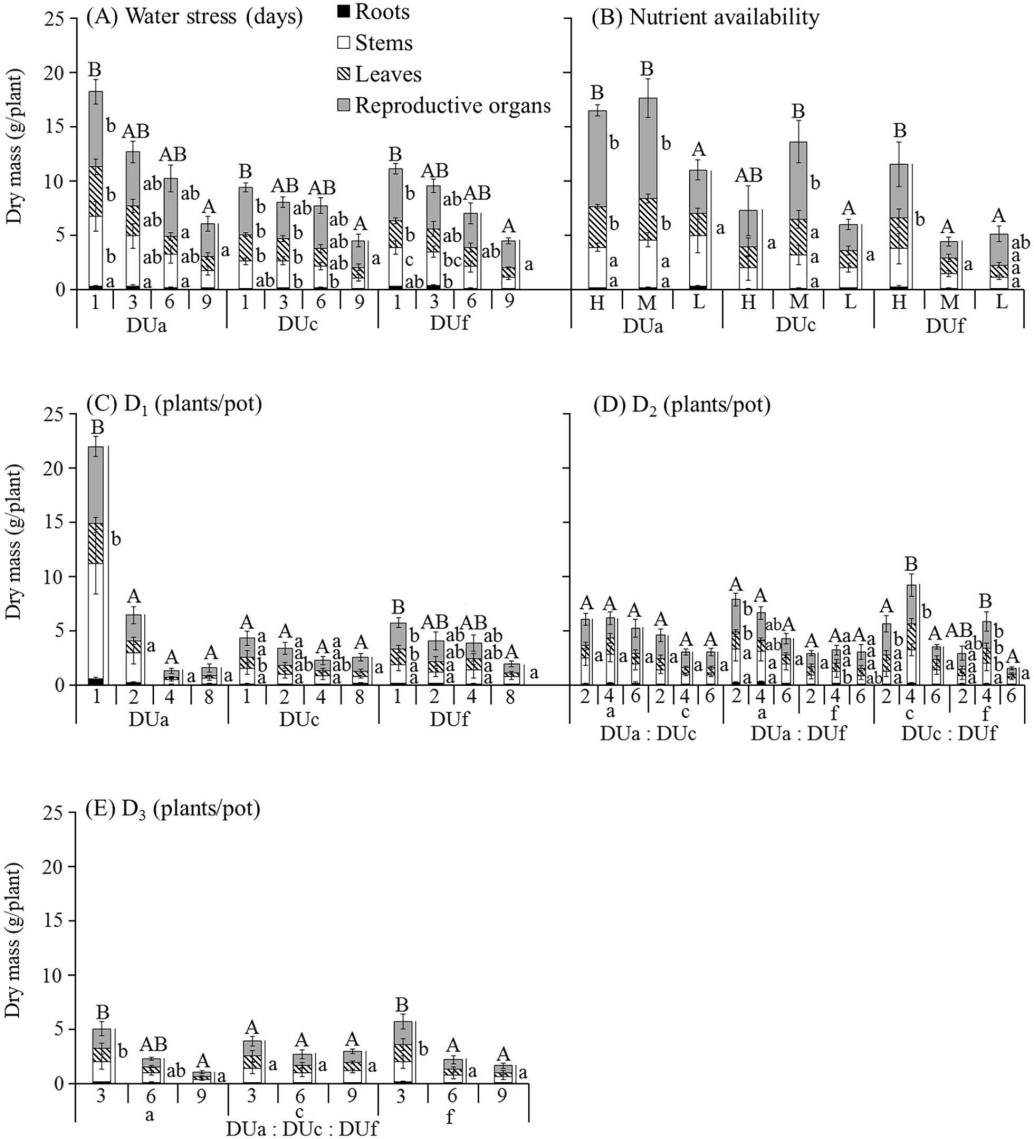
Effect of water stress (**A**), nutrient availability (**B**) and pure (**C**) and mixed (**D**,** E**) density on dry mass of roots, stems, leaves and reproductive organs of *Ceratocarpus arenarius* plants derived from each dispersal unit morph (mean ± 1 s.e.). Only masses ≥ 0.16 g and standard errors ≥ 0.09 g are shown. Different uppercase letters indicate significant differences in total plant mass and different lowercase letters significant differences in mass of roots, stems, leaves and reproductive organs among levels for all treatments of plants from the same dispersal unit morph (*P* < 0.05). DUa, dispersal unit morph a; DUc, dispersal unit morph c; DUf, dispersal unit morph f; H, high; M, moderate; L, low.

**Figure 4:**
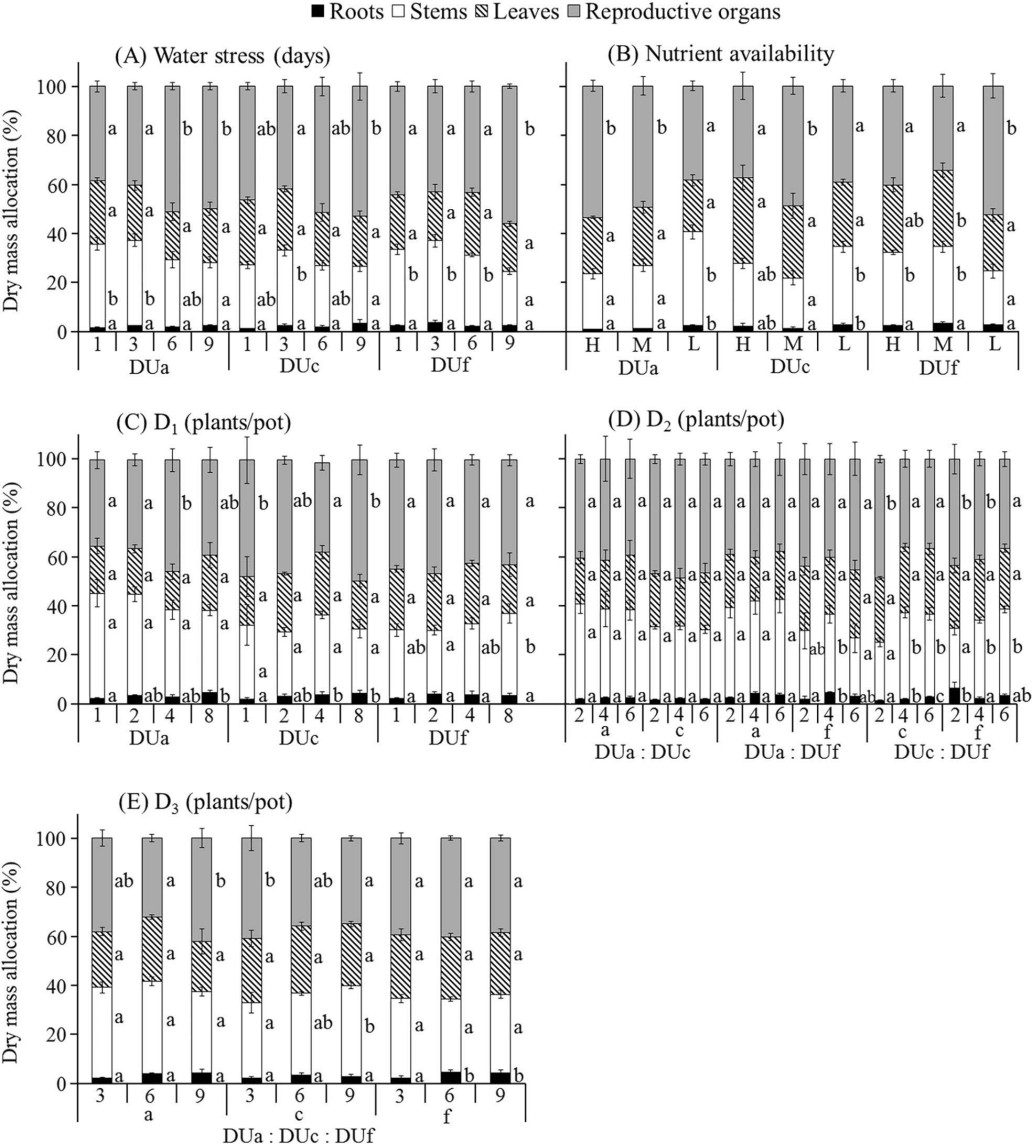
Effect of water stress (**A**), nutrient availability (**B**) and pure (**C**) and mixed (**D**,** E**) density on dry mass allocation to roots, stems, leaves and reproductive organs of *Ceratocarpus arenarius* plants derived from each dispersal unit morph (mean ± 1 SE). Only dry mass allocations ≥ 0.91% and standard errors ≥ 0.66% are shown. Different lowercase letters indicate significant differences in multiple range comparison among levels for all treatments of plants from the same dispersal unit morph (*P* < 0.05). DUa, dispersal unit morph a; DUc, dispersal unit morph c; DUf, dispersal unit morph f; H, high; M, moderate; L, low.

Total plant dry mass and mass of reproductive organs (mass of all dispersal units) were significantly affected by different levels of all treatments in all harvested plants, except D_1_ (Fig. 2A) for plants derived from dispersal unit morph c, D_2(a:c)_ and D_2(a:f)_ (Fig. 2B) for plants from all dispersal unit morphs and D_3_ (Fig. 2C) for plants derived from dispersal unit morph c (Fig. 3). Mass allocation to vegetative and reproductive organs was significantly affected by all treatments (Fig. 4). Allocation to reproductive organs was significantly higher in high than in low water treatment for all harvested plants derived from the three dispersal unit morphs (Fig. 4A). In the nutrient supply treatment, however, the highest allocation to reproductive organs occurred in different nutrient levels for plants derived from the three dispersal unit morphs (i.e. high, moderate and low nutrient supply in plants derived from dispersal unit morphs a, c and f, respectively) (Fig. 4B). In response to different levels of D_1_ (Fig. 4C) and D_3_ (Fig. 4E) treatments, allocation to reproductive organs was significantly higher in high than in low density stress for plants derived from the dispersal unit morph a but nonsignificantly higher in low than in high density stress for plants derived from the dispersal unit morph f. Moreover, allocation to reproductive organs in plants derived from dispersal unit morph c was similar to that of plants derived from dispersal unit morphs a and f in D_1_ and D_3_, respectively. In response to different combinations and levels of the D_2_ treatment, only [D_2(c:f)_] differed significantly in allocation to reproductive organs among the different density levels, which were higher in low than in high density stress for the plants derived from the dispersal unit morphs c and f (Fig. 4D).

### Dispersal unit and fruit production

**Figure 5:**
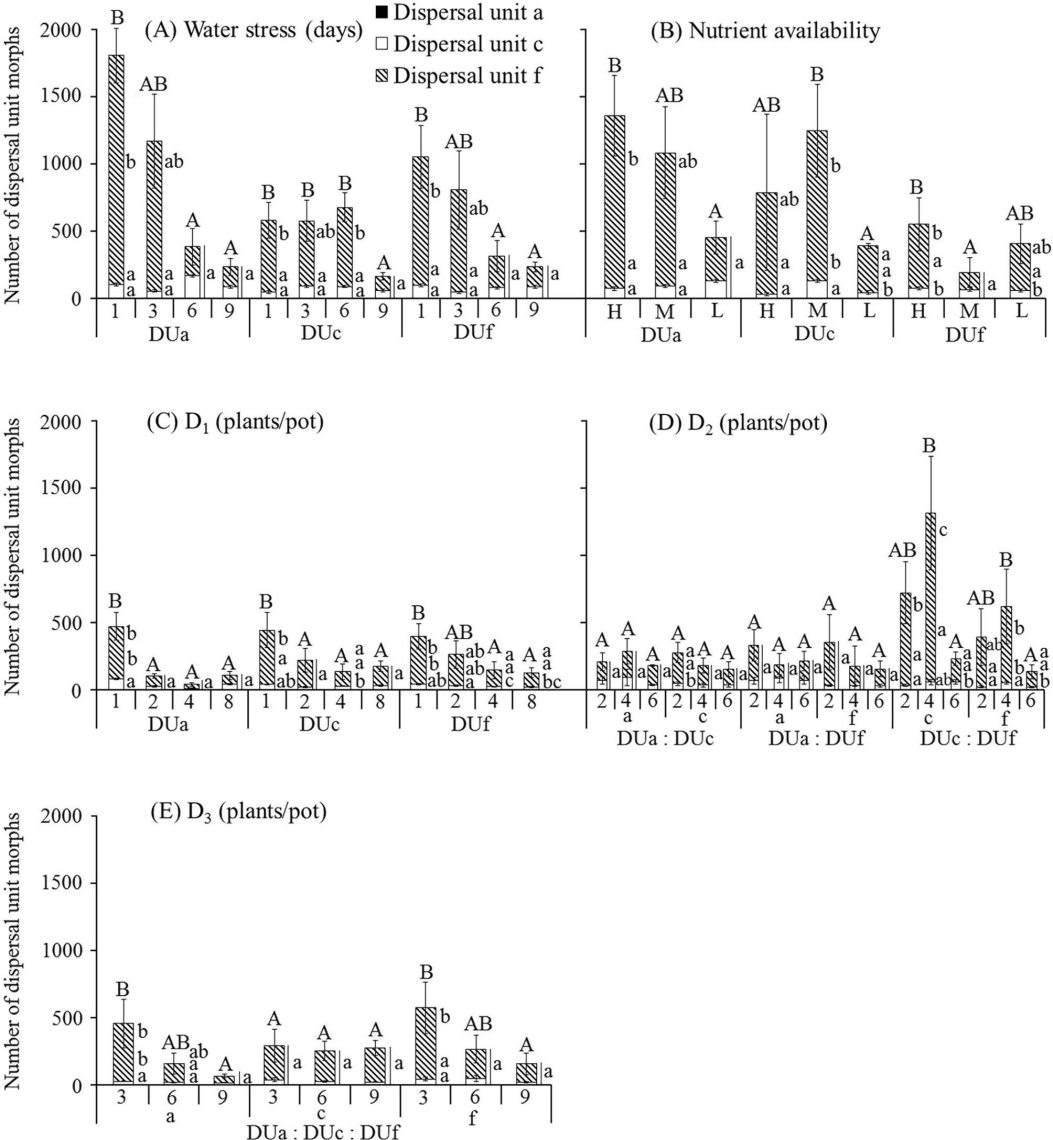
Effect of water stress (**A**), nutrient availability (**B**) and pure (**C**) and mixed (**D**, **E**) density on number of dispersal unit morphs a, c and f of *Ceratocarpus arenarius* plants derived from each dispersal unit morph (mean ± 1 s.e.). Only numbers of each dispersal unit morph ≥ 21.00 and standard errors ≥ 4.34 are shown. Different uppercase letters indicate significant differences in total number of the three dispersal unit morphs and different lowercase letters significant differences in number of each of the three dispersal unit morphs among levels for all treatments of plants from the same dispersal unit morph (P < 0.05). DUa, dispersal unit morph a; DUc, dispersal unit morph c; DUf, dispersal unit morph f; H, high; M, moderate; L, low.

**Figure 6:**
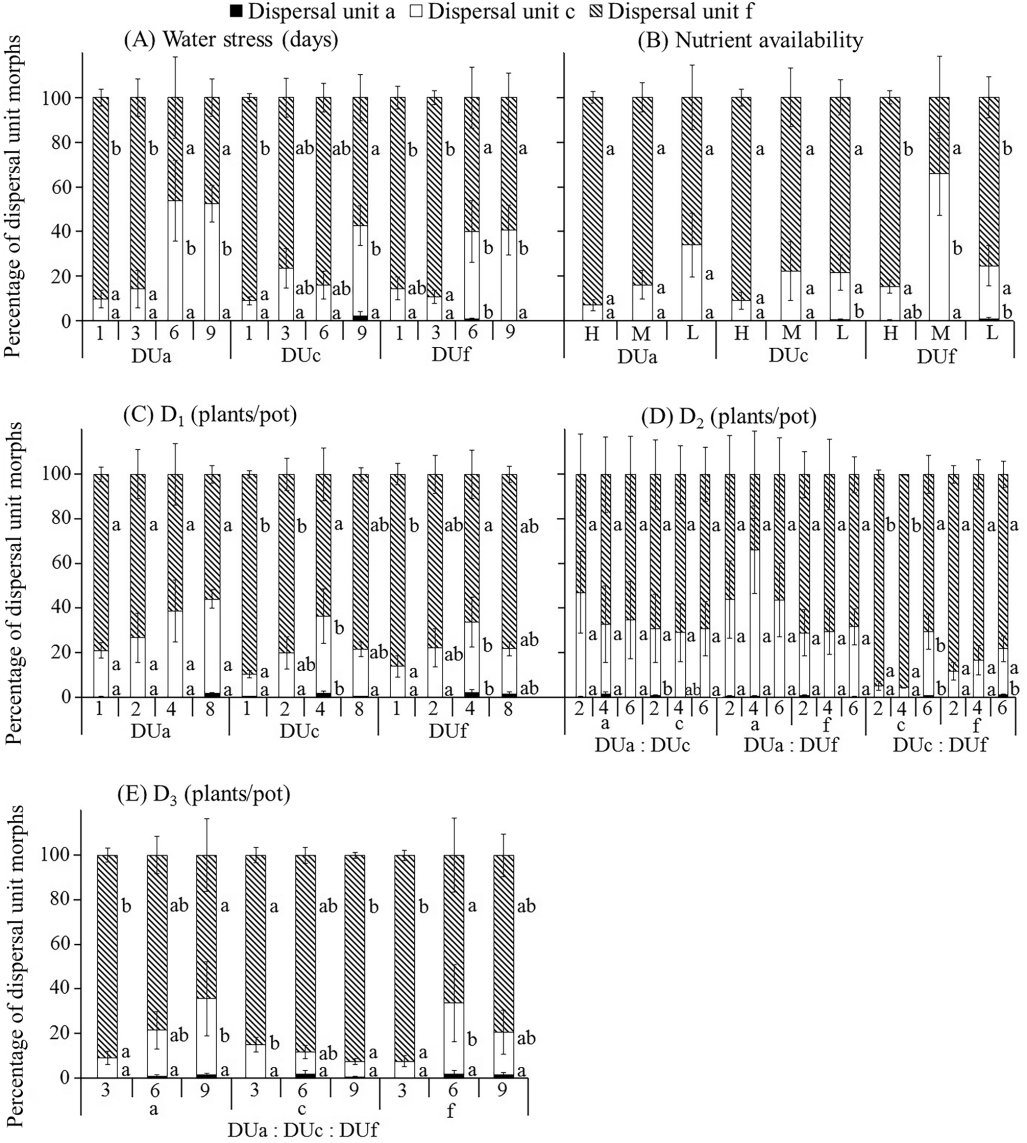
Effects of water stress (**A**), nutrient availability (**B**) and pure (**C**) and mixed (**D**,** E**) density on percentage of dispersal unit morphs a, c and f of *Ceratocarpus arenarius* plants derived from each dispersal unit morph (mean ± 1 s.e.). Only percentages of each dispersal unit morph ≥ 0.51% and standard errors ≥ 0.89% are shown. Different lowercase letters indicate significant difference in multiple range comparison among levels for all treatments of plants from the same dispersal unit morph (*P* < 0.05). DUa, dispersal unit morph a; DUc, dispersal unit morph c; DUf, dispersal unit morph f; H, high; M, moderate; L, low.

With increase in level of stress in which plants derived from the three dispersal unit morphs were grown, total number and total mass of the three dispersal unit morphs and number (Fig. 5), proportion (Fig. 6), mass (Supplementary Fig. S1) and proportion of mass (Supplementary Fig. S2) of dispersal unit morph f decreased. However, number (Fig. 5), proportion (Fig. 6), mass (Supplementary Fig. S1) and proportion of mass (Supplementary Fig. S2) of dispersal unit morph a increased. Trends for number (Fig. 5), proportion (Fig. 6), mass (Supplementary Fig. S1) and proportion of mass (Supplementary Fig. S2) of dispersal unit morph c were the same as those of dispersal unit morphs a or f. In addition, all trends for number (Supplementary Fig. S3) and proportion (Supplementary Fig. S4) for dispersal unit morphs a, c and f with a fruit were almost the same as that of each dispersal unit morph in response to different levels of all treatments.

In the water stress, nutrient availability, D_1_ and D_3_ treatments, two-way ANCOVAs showed that proportion of dispersal unit morphs c and f was significantly affected by water stress and nutrient availability, and that proportion of dispersal unit morph a was significantly affected by nutrient availability and D_1_ (Table 1). Moreover, there were significant differences in proportion of dispersal unit morphs c and f among plants from the three dispersal unit morphs only in the D_1_ treatment (Table 1). In the D_2_ treatment, three-way ANCOVAs showed that only proportion of dispersal unit morphs c and f was significantly affected by combination of any two dispersal unit morphs (Table 2). Correlative analyses also showed that the relationship between total plant mass and proportion of dispersal unit morphs a and c was negatively correlated in most treatments (Supplementary Tables S1–S5). However, the relationship between total plant mass and proportion of dispersal unit morph f was positive in most treatments (Supplementary Tables S1–S5).

**Table 1:** Summary of two-way ANCOVAs showing effects of different levels of water (W), nutrient availability (N), pure (D_1_) or mixed (D_3_) density, plants from the three dispersal unit morphs (M) and their interactions on proportion of each dispersal unit morph from offspring of *Ceratocarpus arenarius*.

Source		Dispersal unit a	Dispersal unit c	Dispersal unit f
W	F	0.49	10.69	10.69
	df	3	3	3
	P	0.70	< 0.05	< 0.05
M	F	0.56	0.58	0.52
	df	2	2	2
	P	0.58	0.56	0.60
W × M	F	1.31	2.02	1.97
	df	6	6	6
	P	0.26	0.07	0.08
N	F	3.74	5.98	5.93
	df	2	2	2
	P	< 0.05	< 0.05	< 0.05
M	F	1.50	2.97	3.05
	df	2	2	2
	P	0.23	0.06	0.06
N × M	F	0.76	2.16	2.09
	df	4	4	4
	P	0.56	0.09	0.09
D_1_	F	3.63	1.79	2.12
	df	3	3	3
	P	< 0.05	0.16	0.11
M	F	0.95	5.86	5.85
	df	2	2	2
	P	0.39	< 0.05	< 0.05
D_1_ × M	F	2.70	1.05	1.24
	df	6	6	6
	P	< 0.05	0.40	0.30
D_3_	F	1.73	0.72	0.98
	df	2	2	2
	P	0.19	0.49	0.38
M	F	0.23	2.50	2.67
	df	2	2	2
	P	0.79	0.09	0.08
D_3_ × M	F	0.43	2.14	2.32
	df	4	4	4
	P	0.79	0.09	0.07

There was no significant effect of initial seedling size, which was used as a covariate, on any of the variables in the analyses (data not shown).

**Table 2:** Summary of three-way ANCOVAs showing effects of different levels of mixed density (D_2_), plants from the three dispersal unit morphs (M), combination type of any two dispersal unit morphs (C) and their interactions on proportion of each dispersal unit morph from offspring of *Certaocarpus arenarius*.

Source		Dispersal unit a	Dispersal unit c	Dispersal unit f
D_2_	F	0.01	1.05	1.56
	df	2	2	2
	P	0.99	0.36	0.22
M	F	0.79	0.40	0.63
	df	2	2	2
	P	0.46	0.67	0.53
C	F	0.47	4.99	3.43
	df	2	2	2
	P	0.63	< 0.05	< 0.05
D_2_ × M	F	2.48	0.13	0.34
	df	4	4	4
	P	0.05	0.97	0.85
D_2_ × C	F	4.18	0.67	0.35
	df	4	4	4
	P	< 0.05	0.61	0.84
M × C	F	0.77	1.20	1.70
	df	4	4	4
	P	0.38	0.28	0.20
D_2_ × M × C	F	1.54	3.51	4.22
	df	8	8	8
	P	0.22	< 0.05	< 0.05

There was no significant effect of initial seedling size, which was used as a covariate, on any of the variables in the analyses (data not shown).

## Discussion

The most important findings of our study were that most levels of abiotic (soil water content and nutrient supply) and biotic (density) treatments caused a decrease in number and mass of the uppermost dispersal unit (morph f) of *C. arenarius* but an increase or no change in number and mass of the lowermost dispersal unit (morph a). Moreover, a striking result of the present study is that trends for number and mass of dispersal unit morph c were the same as those of dispersal unit morphs a or f, depending on level of treatment. Further, in most stress conditions there was a downward shift in proportion of mass and number for dispersal unit morph f, while proportion of number and mass for dispersal unit morph a increased. Thus, our hypothesis that increasing stress causes a decrease in production of dispersal units with high dispersal and low dormancy in favor of dispersal units with high dormancy and no dispersal is supported.

Both water and density treatments caused a significant shift in proportion of dry mass allocated to vegetative and reproductive components in the diaspore-dimorphic species *Diptychocarpus strictus*^18^ and *Lappula duplicicarpa*^19^ . In particular, the proportion of dry mass allocated to reproductive organs increased in stressed plants, while the proportion of dry mass allocated to vegetative organs in these two species and also in *C. arenarius* correspondingly decreased. In *C. arenarius*, however, variation in reproductive mass (CV 89.22%, 92.45% and 86.45% in plants derived from dispersal unit morphs a, c and f, respectively) was higher than that of total plant mass (CV 87.78%, 82.35% and 85.60%, respectively).

The conditions to which the parent plant is exposed influence the number^16,23^, mass^15,36^ and size^23,37^ of diaspores produced. The proportion of diaspore morphs produced by heteromorphic plants [i.e. (HDi-LDo)/(LDi-HDo)] morph ratio is a response to the environmental conditions under which the plant is grown. If the ratio is > 1.00, production of (HDi-LDo) diaspore morphs is higher than that of (LDi-HDo) diaspore morphs, and if the ratio is < 1.00, production of (HDi-LDo) diaspore morphs is lower than that of (LDi-HDo) diaspore morphs. Thus, a decrease in the (HDi-LDo)/(LDi-HDo) morph ratio has been demonstrated for increasing soil water^18,21^, nutrient^15,36,37^, density^16,19^ and herbivory^18^ stress. Likewise, in the polymorphic diaspore species *C. arenarius* soil water, nutrient and density stress caused a decrease in proportion of dispersal unit morph f (HDi-LDo) with increase in intensity of all the three stresses, whereas proportion of dispersal unit morph a (LDi-HDo) increased with increase in intensity of all the three stresses. Moreover, proportion of dispersal unit morph c was the same as that of dispersal unit morph f (i.e. decreased) or a (i.e. increased) with increase in intensity of stress. Overall, then, the (HDi-LDo)/(LDi-HDo) ratio decreased with increasing stress in *C. arenarius* (Fig. 6; Table 3). In the heterodiasporous species *Lappula duplicicarpa*^19^, *Atriplex aucheri*^38^ and *Heterosperma pinnatum*^26^, the dispersal unit morph number ratio can vary, depending on the environment conditions under which the mother plants are grown. This diaspore number ratio also varies in the amphicarpic sensu lato species *Catananche lutea*^22^, *Emex spinosa*^39^ and *Gymnarrhena micrantha*^40^, the amphicarpic sensu stricto species *Amphicarpum purshii*^41,42^ and *Mimulus nasutus* with aerial chasmogamous and cleistogamous fruits^43^. In amphicarpic species, aerial diaspores may not be produced if growth conditions are highly stressful; thus, the aerial diaspore/subterranean diaspore ratio can be zero^41^. In the grass *Bromus unioloides*, the aerial CH/CL ratio varied with photoperiod and soil moisture conditions of the mother plants and ranged from 0 to 100%^44^. In *H. pinnatum*, the proportion of achenes with awns increased with decrease in R_0_ (fitness), i.e. increase in stress, and was nearly 1.0 in environments in which R_0_ < 1.0, i.e. a negative correlation between fitness and proportion of awned achenes. For this species, the authors reported that (depending on stress level) individuals can produce any ratio combination of dimorphic achenes from 0 to 1.0^45^. However, even under high stress the (HDi-LDo)/(LDi-HDo) ratio was not zero for *C. arenarius* or for the heterodiasporous species *Diptychocarpus strictus*^18^ and *Lappula duplicicarpa*^19^.

**Table 3:** Variation in the ratio of dispersal unit morphs a, c and f of *Ceratocarpus arenarius* plants derived from each dispersal unit morph grown under nonstressful and stressful conditions.

Treatments	Plant origin	Ratio of dispersal units (a + c): f	Plants under no stress (i.e. best condition)		Plants under highest stress (i.e. worst condition)	
			Total number of dispersal units	Ratio of dispersal units (a: c: f)	Total number of dispersal units	Ratio of dispersal units (a: c: f)
Water stress	Dispersal unit a	↑	1795.13	0.04: 5.74 : 94.23	234.26	0.06: 36.82: 63.13
	Dispersal unit c	↑	578.13	0.00: 8.13: 91.87	164.88	0.38: 35.63: 63.99
	Dispersal unit f	↑	1046.00	0.07: 9.36: 90.58	233.76	0.00: 37.06: 62.94
Nutrient availability	Dispersal unit a	↑	1356.60	0.00: 5.34: 94.66	450.38	0.08: 29.03: 70.88
	Dispersal unit c	↑	788.00	0.00: 3.65: 96.35	388.86	0.33: 10.91: 88.76
	Dispersal unit f	↑	550.75	0.14: 13.30: 86.56	405.38	0.25: 13.78: 85.97
D_1_	Dispersal unit a	↑	472.01	0.09: 17.01: 82.90	108.60	0.92: 38.67: 60.41
	Dispersal unit c	↑	444.25	0.17: 9.12: 90.71	177.50	0.28: 19.15: 80.56
	Dispersal unit f	↑	398.67	0.08: 10.83: 89.09	128.40	0.93: 16.82: 82.24
D_2_						
D_2(a: c)_	Dispersal unit a	↓	207.40	0.29: 33.65: 66.06	179.80	0.11: 20.02: 79.87
	Dispersal unit c	↑	272.25	0.37 : 16.25: 83.38	151.60	0.13: 23.75: 76.12
D_2(a: f)_	Dispersal unit a	↑	325.60	0.25: 20.95: 78.81	210.20	0.10: 34.25: 65.65
	Dispersal unit f	↑	347.75	0.22: 8.55: 91.23	153.80	0.13: 19.64: 80.23
D_2(c: f)_	Dispersal unit c	↑	708.20	0.00: 4.72: 95.28	228.40	0.53: 24.34: 75.13
	Dispersal unit f	↑	386.20	0.05: 5.96: 93.99	133.20	1.05: 14.71: 84.23
D_3_	Dispersal unit a	↑	446.40	0.09: 5.15: 94.76	61.40	1.63: 18.89: 79.48
	Dispersal unit c	↓	285.00	0.28: 11.72: 88.00	268.40	0.45: 6.04: 93.52
	Dispersal unit f	↑	562.20	0.11: 7.19: 92.71	152.20	0.79: 10.12: 89.09

Ratio of dispersal unit (a + c): f increased (↑), decreased (↓).

The effect of dispersal unit morph on CV of dispersal unit number and mass varied by treatment, with dispersal unit morph f having the highest CV for number (range 1.24 to 1.62) and mass (range 1.10 to 1.53) and dispersal unit morph a the lowest CV for number (range 0.25 to 0.41) and mass (range 0.49 to 0.76). The CV for dispersal unit morph c for number (range 1.04 to 1.26) and mass (range 0.94 to 1.18) was between those of dispersal unit morphs a and f. The effects of dispersal unit morph on the CV of dispersal units with a fruit varied by treatment in the same manner as that of the dispersal unit morphs, i.e. f > c > a.

The shift in proportion of heteromorphic diaspores is of ecological significance in the dispersal and germination stages of a plant’s life history^2,46^ . In stressful and nonstressful environmental conditions, dispersal unit morph a of *C. arenarius* is a low-risk phenotype that germinates in a proven site occupied by the mother plant, and dispersal unit morph f is a high-risk phenotype that explores new habitats away from the mother plant. However, as expected, both number and proportion of morph f were higher in nonstressful than stressful situations. These results are in agreement with those of Venable et al. who found that populations of *H. pinnatum* with a higher proportion of central achenes with awns (high-dispersal, low-dormancy achenes) occurred in sites where precipitation was relatively high^26^. Thus, more high-risk diaspores are available for colonization of sites away from the maternal plants of *C. arenarius* in nonstressful than in stressful conditions. Consequently, with an increase in favorable conditions for growth and survival of her offspring the mother plant devotes proportionally more resources to the high-risk morph.

As such, then, production of dispersal unit morph a is a “timid” strategy for ensuring reproduction, while production of dispersal unit morph f is a “bold” strategy^47,48^. Moreover, production of dispersal unit morph c is a transitional type between “timid” and “bold” strategies. However, regardless of environmental conditions (stressful vs. nonstressful) presumably some degree of bet-hedging capability was retained: under no environmental condition was there a complete shift to production of only one morph. This retention of a (HDi-LDo)/(LDi-HDo) morph ratio greater than zero under very stressful environmental conditions also occurs in the heterodiasporous plants *Atriplex sagittata*^15,36^, *Leontodon saxatilis*^16^, *Diptychocarpus strictus*^18^ and *Lappula duplicicarpa*^19^.

Variable diaspore functions in heteromorphic species usually are explained in terms of bet-hedging^2^, an adaptation to highly variable and unpredictable environments in time or space^14,49^. The success of *C. arenarius* in the cold desert of Central Asia might be, at least in part, attributed to production of a range of variation in dispersal unit morphs and the ability to shift the dispersal unit proportion under changing environmental conditions.

## Materials and methods

### Dispersal unit collection and field site description

Freshly-matured dispersal units with khaki-coloured persistent bracteoles (Fig. 1) were collected from many individuals in a natural population of several thousand *C. arenarius* plants growing on desert sand dunes in the vicinity of Fukang city on the southern edge of the Junggar Basin of Xinjiang Province, China (44°22′N, 87°53′E, 438 m a. s. l.) on 3 October 2016^35^. In the laboratory, dispersal units from individual plants were separated from other plant material and divided into (1) those at soil surface (basicarps) and (2) each of five aerial diaspore morphs in the gradient of dispersal units above the soil surface (Fig. 1)^33^. Dispersal unit morphs a, c and f were bulked separately and stored in paper bags at room conditions (16–30 °C, 10–40% RH) until used in experiments.

The area of the Junggar Basin where the dispersal units were collected has a temperate continental climate. Mean annual temperature is 6.6 °C, and average annual precipitation (including rain and snow) is 160 mm, about two-thirds of which falls in spring and summer, and the snow that falls in winter begins to melt in March or April^50^. Annual potential evaporation is > 2000 mm^51,52^. Additionally, rainfall is highly variable among seasons and years, but generally, rainfall is higher in spring than in autumn^53,54^. Further, water from snowmelt increases water availability in spring.

### Experimental design

Our observation in the experimental garden and in a diversity of habitat types in the field, levels of soil moisture, nutrients and competition (density) are the important contributors to the phenotypic responses (plant size and dispersal unit production) of *C. arenarius* plants.

To obtain seedlings from dispersal unit morphs a, c and f, on 4 April 2017, 1,600 isolated fruits (i.e. dispersal units with their bracteoles removed) of each dispersal unit morphs a, c and f were incubated in 0.5 mmol·L^−1^ GA_3_ solutions at 25/15 °C in light/dark (12/12 h) for five days. Then, during 10–15 April 2017, 3,720 seedlings (10 seedlings per pot × three dispersal unit morphs × 124 pots) that germinated on the same day were transplanted into pots (20 cm deep and 24 cm in diameter) with drainage holes in the bottom. The pots were filled with a mixture of 70% grey desert soil and 30% desert sand; sand was added to improve soil aeration and drainage. Seedlings were exposed to near-natural temperatures in a metal framehouse with no heating or cooling with sides open, in the experimental garden on the campus of Xinjiang Agricultural University in Urümqi, which is located near the southern edge of the Junggar Basin^19,55^. Before the beginning of each treatment (see below), the soil was watered daily to field capacity. To prevent variation in initial seedling size, seedlings of the same size in each pot were kept (i.e. one plant per pot), and the others were removed from the pots and discarded, except in the density treatment with ≥ 1 to 9 seedlings per pot (see below)^18^. A factorial design was used in the soil water content, nutrient supply and density treatments, which were initiated when plants were in the four-leaf rosette stage (during 25–30 April 2017, 15 days after transplanting)^19^. In addition, the number of individuals surviving from the four-leaf rosette stage to reproduction was monitored. Fifty to 62.5% of seedlings from the three dispersal unit morphs at high levels of nutrient supply and ≥ 90% of seedlings from the other treatments survived and reproduced; the experiment ended on 3 October 2017.

Each treatment was an independent test. While one factor was manipulated, the others were kept at high or moderate levels to prevent limitation of plant growth. That is, the soil was kept moist except in the soil water content treatment, one plant per pot except in the density treatment and no nutrient supply added except in the nutrient supply treatment. Weeds were removed from the pots as needed to prevent competition from them^18,19^.

#### Soil water content

Four moisture levels were applied to the plants: watered to field capacity every day (high water supply, i.e. the lowest stress), every three days, every six days or every nine days (i.e. the highest stress)^18^. Evidence for water stress was shown by the smaller size of plants in the 9-day than 6-day and in the 6-day than in the 3-day watering treatment.

#### Nutrient supply

Treatment levels for the nutrient gradient were low (no fertilizer added, only tap water; control) (i.e. the highest stress), moderate [3.0 g of 15:15:15 (N: P: K) per liter of water] and high [6.0 g of 15:15: 15 (N: P: K) per liter of water] (i.e. the lowest stress). For each concentration, each pot received 1.0 L of treatment solution once every two weeks until senescence. Once every two weeks, soil in each pot was flushed with 2.0 L of tap water to prevent accumulation of nutrients. Then, 1.0 L of the treatment solution or tap water (control) was added to the soil in each pot; it was applied directly to the base of each plant^18^. Evidence for nutrient stress was shown by smaller size of plants in the 0 than in the 3 g nutrient addition and in the 3 g than in the 6 g nutrient addition.

#### Density

Density experiments were carried out using pure (D_1_) and mixed (D_2_ and D_3_) stands. D_1_ was used to study the effect of density stress on vegetative and reproductive dry mass of plants from the different dispersal unit morphs. Four levels of intramorph D_1_ were used: (1) one plant in the pot (target plant) (i.e. the lowest stress), (2) one target plant and one other plant from the same morph, (3) target plant surrounded by three plants from the same morph and (4) target plant surrounded by seven plants from the same morph (the highest stress) (Fig. 6A).

Plants from two dispersal unit morphs were combined in a 1:1 ratio at three total densities [two (i.e. the lowest stress), four and six (i.e. the highest stress) plants per pot]. Plants from the two dispersal units were planted alternately, and combinations of plants from any two of the three dispersal unit morphs were used: (1) dispersal units a and c [D_2(a:c)_], (2) dispersal units a and f [D_2(a: f)_] and (3) dispersal units c and f [D_2(c: f)_] (Fig. 6B).

Competition among plants from the three dispersal unit morphs (D_3_) was tested in a 1:1:1 ratio at three total densities [three (i.e. the lowest stress), six and nine (i.e. the highest stress) plants per pot] (Fig. 6C).

## Measurements

Each level of soil water content treatment was replicated eight times (eight plants were harvested to measure all indices at maturity), and nutrient supply treatment was replicated eight times at low and intermediate levels and four times at high levels. Except for eight replicates of one plant per pot in the D_1_ treatment, the other levels of D_1_ and each level of D_2_ and of D_3_ was replicated five times. We harvested each plant during 6–22 September 2017, when there was a total lack of chlorophyll (plant death). When all dispersal units were mature, plants in each treatment were excavated to a soil depth of 15 cm, and roots were gently washed free of soil^19^.

### Dry mass accumulation and allocation

At harvest, plants in each treatment were collected separately and divided into root, stem, leaf and reproductive organs [i.e. including all dispersal unit morphs and their glochids (if present) and trichomes on bracts] and oven-dried to constant mass at 80 °C for 48 h in paper bags. Once dry, all parts were weighed using an electronic balance (0.0001 g). Vegetative mass was calculated as sum of mass of roots, stems and leaves. Total mass is vegetative mass plus mass of reproductive organs. Allocation to roots, stems, leaves and reproductive organs was calculated by dividing mass of each organ by total mass and then multiplying by 100^18^. Additionally, reproductive mass was expressed as coefficient of variation (CV = standard deviation/mean) in plants derived from each dispersal unit morph.

### Dispersal unit and fruit production

#### Number

Number of dispersal unit morphs a, c and f on the plant in each replicate at different levels in the treatments was recorded. Total number of dispersal units per individual was determined by summing number of all dispersal unit morphs per individual. By randomly sampling and making observations, we found that if there is a fully developed fruit/seed in a dispersal unit the fruit is inflated and can be seen clearly through the bracteoles under a fluorescent light. However, if a fruit/seed is not present in a dispersal unit the bracteoles are not inflated. Thus, the number of each kind of dispersal unit morphs (with and without a fruit) on each plant was counted using the observation method described above. Percentage of each of the three dispersal unit morphs (with + without a fruit) (NP_j_) and percentage of each of the three morphs with a fruit (NP_j_ʹ) was calculated as NP_j_ = (ND_j_ / NT) × 100 and NP_j_ʹ = (ND_j_ʹ/ NTʹ) × 100, respectively, where ND_j_ is total number of each of the three dispersal unit morphs (with + without a fruit) and ND_j_ʹ number of each of the three dispersal unit morphs with a fruit, NT total number of the three dispersal unit morphs and NTʹ total number of the three dispersal unit morphs with a fruit per individual and j the given dispersal unit morph (a, c or f).

#### Mass

Each dispersal unit morph per individual was weighed using the electronic balance (0.0001 g). Mass percentage of each of the three dispersal unit morphs per individual was calculated by dividing mass of each dispersal unit morph by total mass of the three dispersal unit morphs and then multiplying by 100.

Additionally, number and mass of dispersal unit a, c and f morphs was expressed as CV (i.e. standard deviation/mean) in plants derived from each dispersal unit morph.

### Statistical analysis

A one-way ANOVA was used to analyze differences in plant dry mass; allocation of dry mass to roots, stems, leaves and reproductive organs; number, proportion, mass and proportion of mass of each dispersal unit morph a, c and f; total number and mass of the three dispersal unit morphs; and number and proportion of each dispersal unit morph with a fruit per individual among levels in each treatment. Data were arcsine (percentage data) or log_10_ (other data) transformed as needed before analysis to approximate a normal distribution and homogeneity of variance to fulfill assumptions of a one-way ANOVA. If variance of transformed data was still not homogenous, treatment differences in these variables were assessed using the Kruskal–Wallis non-parametric test. Tukey’s HSD test was performed for multiple comparisons to determine significant differences among treatments^56^.

Two-way ANCOVAs or three-way ANCOVAs were used to determine whether proportion of each dispersal unit morph differed among levels in each treatment. For “soil water content”, “nutrient supply”, “D_1_” and “D_3_” treatments in the two-way ANCOVAs, plants from the three dispersal unit morphs and treatment (soil water content, nutrient supply, D_1_ and D_3_) were considered fixed effects, and all interaction terms were included. For the “D_2_” treatment in the three-way ANCOVAs, plants from the three dispersal unit morphs, treatment (levels of D_2_) and combination with any two morphs of the three dispersal unit morphs [D_2(a:c)_, D_2(a:f)_ and D_2(c:f)_] were considered fixed effects, and all interaction terms were included. Total mass was used as a covariate to minimize the effects of variation in plant size on the proportion in the analyses. Pearson correlations were used to determine the relationships between proportion of any two of the three dispersal unit morphs and between mass of total plant and proportion of each dispersal unit morph in each treatment (Supplementary Tables S1–S5). All data analyses were performed with the software SPSS 19.0 (SPSS Inc, Chicago, IL, U.S.A.). Values are means ± 1 s.e.

## Supplementary information

Supplementary figures

Supplementary tables

Supplementary information
