# Supplementary material for: Phenotypic plasticity in diaspore production of a amphi-basicarpic cold desert annual that produces polymorphic diaspores
Source: Sci Rep. 2020 Jul 7;10:11142. doi: 10.1038/s41598-020-67380-0 (PMC7341796; doi:10.1038/s41598-020-67380-0)

Phenotypic plasticity in diaspore production of a amphi-basicarpic cold desert annual that produces polymorphic diaspores

Lu Gan[1], Juanjuan Lu[1]*, Jerry M. Baskin[1,2], Carol C. Baskin[1,2,3] and Dunyan Tan[1]*

[1]College of Grassland and Environment Sciences, Xinjiang Agricultural University, Urümqi, China

[2]Department of Biology, University of Kentucky, Lexington, KY, USA

[3]Department of Plant and Soil Sciences, University of Kentucky, Lexington, KY, USA

* E-mail: juanjuan124391@163.com or tandunyan@163.com

Short-title: Phenotypic plasticity in an amphi-basicarpic species

Supplementary Fig. S1

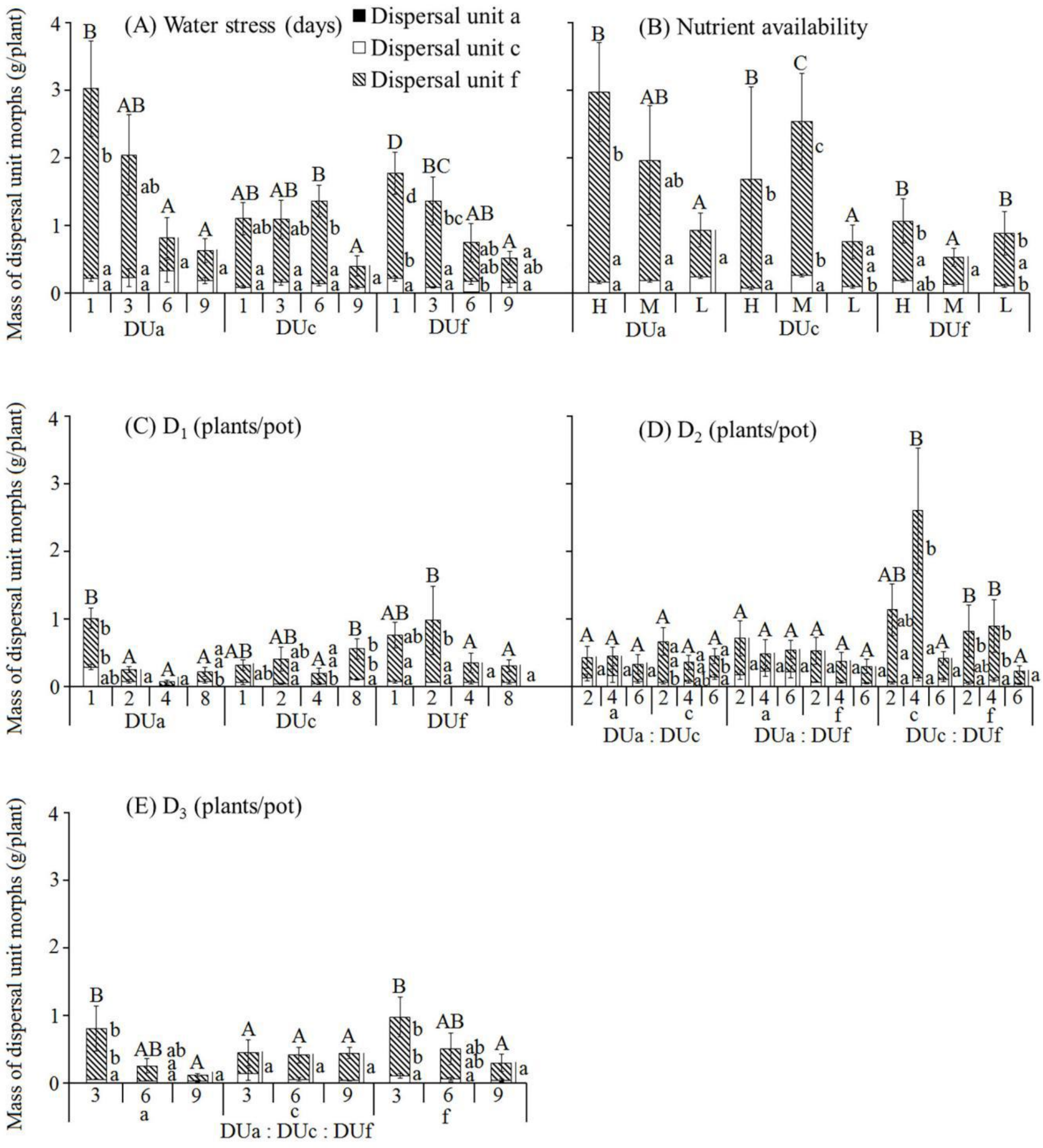

Supplementary Fig. S2

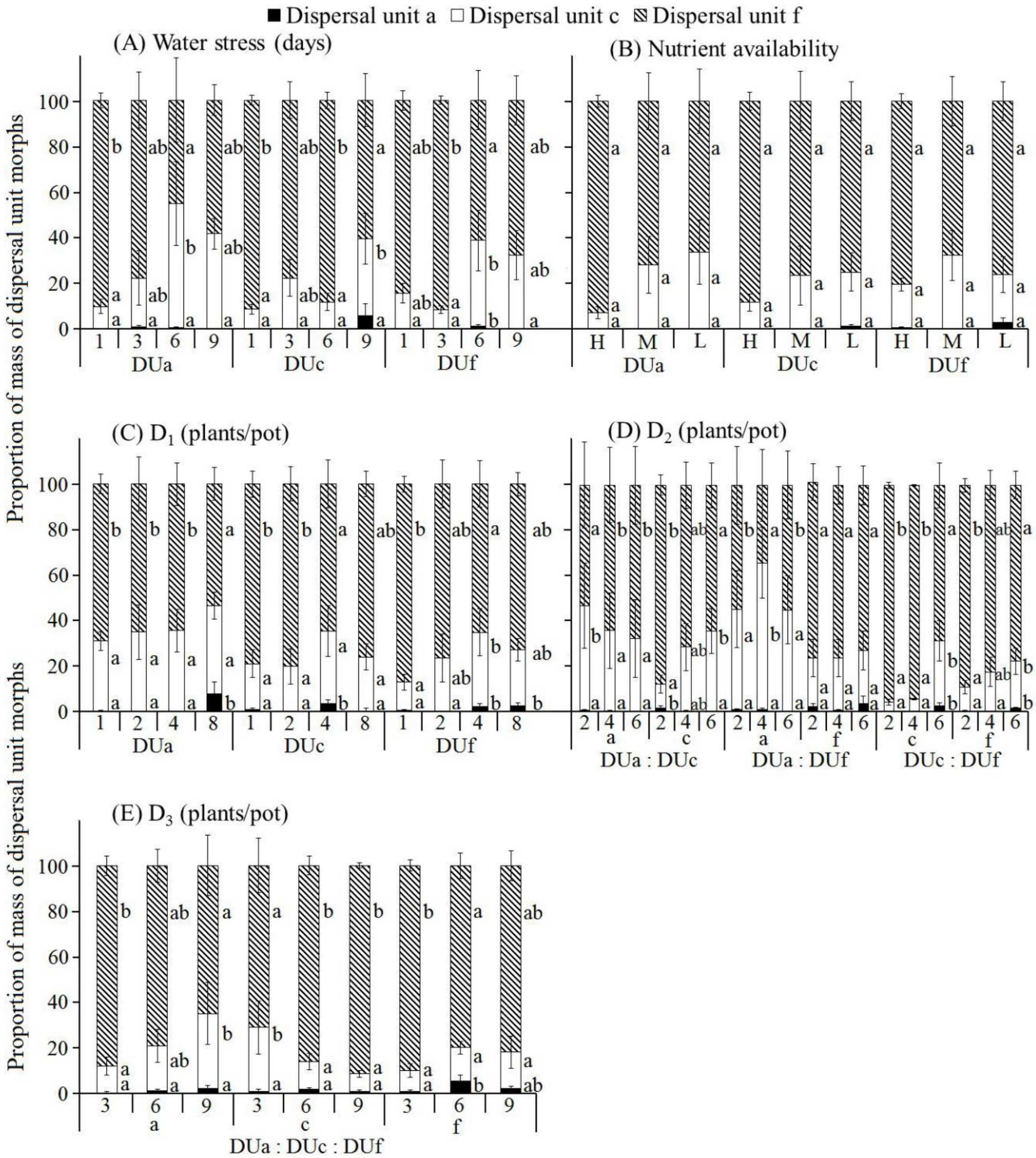

Supplementary Fig. S3

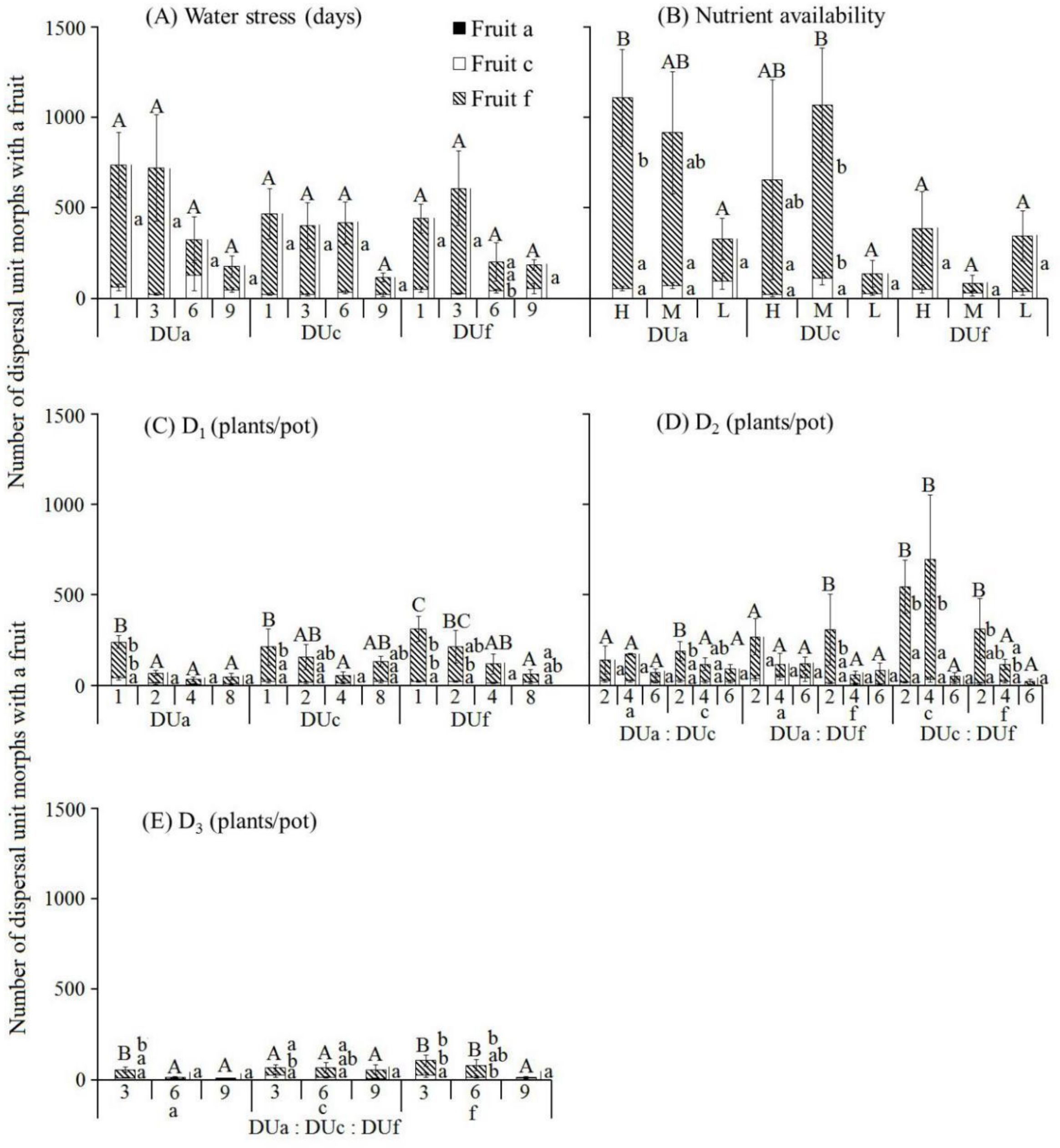

Supplementary Fig. S4

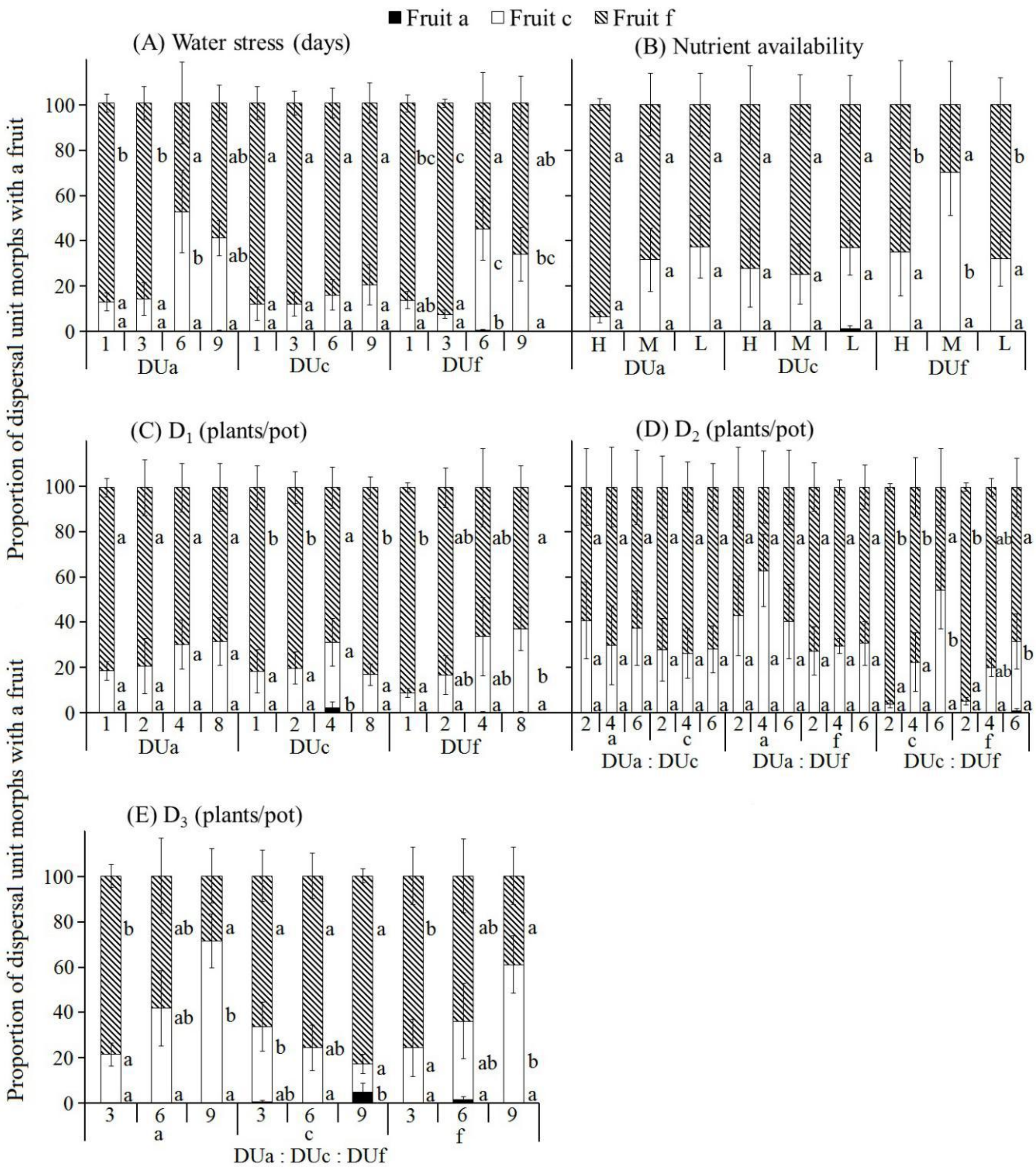

Supplement: Supplementary file 1 — Supplementary figures [file 41598_2020_67380_MOESM1_ESM.pdf]
