# Supplementary material for: Phenotypic plasticity in diaspore production of a amphi-basicarpic cold desert annual that produces polymorphic diaspores
Source: Sci Rep. 2020 Jul 7;10:11142. doi: 10.1038/s41598-020-67380-0 (PMC7341796; doi:10.1038/s41598-020-67380-0)
Supplement: Supplementary file 2 — Supplementary tables [file 41598_2020_67380_MOESM2_ESM.pdf]

Table S1: Pearson correlations between proportion of any two of the three dispersal unit morphs and between mass of total plant and proportion of each dispersal unit morph for *Ceratocarpus arenarius* plants derived from the three dispersal unit morphs in different levels of water stress. N = 62, 60 and 60 for each correlation of plants from dispersal unit a, c and f morphs, respectively. * $P < 0.05$; ** $P < 0.01$.

Plant origin	Characters	Proportion of dispersal unit a morph	Proportion of dispersal unit c morph	Proportion of dispersal unit f morph
Plants from dispersal unit a morph	Proportion of dispersal unit a morph	1.00		
	Proportion of dispersal unit c morph	0.26	1.00	
	Proportion of dispersal unit f morph	-0.27	-1.00**	1.00
	Mass of total plant	-0.03	-0.36*	0.36*
Plants from dispersal unit c morph	Proportion of dispersal unit a morph	1.00		
	Proportion of dispersal unit c morph	0.53**	1.00	
	Proportion of dispersal unit f morph	-0.62**	-0.99**	1.00
	Mass of total plant	-0.38*	-0.40*	0.42*
Plants from dispersal unit f morph	Proportion of dispersal unit a morph	1.00		
	Proportion of dispersal unit c morph	-0.05	1.00	
	Proportion of dispersal unit f morph	0.03	-1.00**	1.00
	Mass of total plant	-0.22	-0.22	0.23

Table S2: Pearson correlations between proportion of any two of the three dispersal unit morphs and between mass of total plant and proportion of each dispersal unit morph for *Ceratocarpus arenarius* plants derived from the three dispersal unit morphs in different levels of nutrient supply. N = 40, 36 and 32 for each correlation of plants from dispersal unit a, c and f morphs, respectively. ** P < 0.01.

Plant origin	Characters	Proportion of dispersal unit a morph	Proportion of dispersal unit c morph	Proportion of dispersal unit f morph
Plants from dispersal unit a morph	Proportion of dispersal unit a morph	1.00		
	Proportion of dispersal unit c morph	-0.02	1.00	
	Proportion of dispersal unit f morph	0.02	-1.00**	1.00
	Mass of total plant	-0.21	0.01	-0.01
Plants from dispersal unit c morph	Proportion of dispersal unit a morph	1.00		
	Proportion of dispersal unit c morph	0.06	1.00	
	Proportion of dispersal unit f morph	-0.08	-1.00**	1.00
	Mass of total plant	-0.30	-0.29	0.29
Plants from dispersal unit f morph	Proportion of dispersal unit a morph	1.00		
	Proportion of dispersal unit c morph	-0.02	1.00	
	Proportion of dispersal unit f morph	-0.01	-1.00**	1.00
	Mass of total plant	-0.17	-0.11	0.12

Table S3: Pearson correlations between proportion of any two of the three dispersal unit morphs and between mass of total plant and proportion of each dispersal unit morph for *Ceratocarpus arenarius* plants derived from the three dispersal unit morphs in different levels of pure density (D1). N = 44, 36 and 42 for each correlation of plants from dispersal unit a, c and f morphs, respectively. ** $P < 0.01$.

Plant origin	Characters	Proportion of dispersal unit a morph	Proportion of dispersal unit c morph	Proportion of dispersal unit f morph
Plants from dispersal unit a morph	Proportion of dispersal unit a morph	1.00		
	Proportion of dispersal unit c morph	0.08	1.00	
	Proportion of dispersal unit f morph	-0.15	-.998**	1.00
	Mass of total plant	-0.17	-0.29	0.30
Plants from dispersal unit c morph	Proportion of dispersal unit a morph	1.00		
	Proportion of dispersal unit c morph	0.04	1.00	
	Proportion of dispersal unit f morph	-0.11	-1.00**	1.00
	Mass of total plant	-0.21	-0.62**	0.63**
Plants from dispersal unit f morph	Proportion of dispersal unit a morph	1.00		
	Proportion of dispersal unit c morph	0.03	1.00	
	Proportion of dispersal unit f morph	-0.15	-0.99**	1.00
	Mass of total plant	-0.23	-0.55**	0.57**

Table S4: Pearson correlations between proportion of any two of the three dispersal unit morphs and between mass of total plant and proportion of each dispersal unit morph for *Ceratocarpus arenarius* plants derived from the three dispersal unit morphs in different levels of mixed density (D2). N = 30 for each correlation of plants from each dispersal unit morph. * P < 0.05; ** P < 0.01.

Combination type	Plant origin	Characters	Proportion of dispersal unit a morph	Proportion of dispersal unit c morph	Proportion of dispersal unit f morph
D2 (a:c)	Plants from dispersal unit a morph	Proportion of dispersal unit a morph	1.00		
		Proportion of dispersal unit c morph	-0.16	1.00	
		Proportion of dispersal unit f morph	0.12	-1.00**	1.00
		Mass of total plant	-0.40	0.27	-0.25
	Plants from dispersal unit c morph	Proportion of dispersal unit a morph	1.00		
		Proportion of dispersal unit c morph	0.45	1.00	
		Proportion of dispersal unit f morph	-0.47	-1.00**	1.00
		Mass of total plant	0.03	-0.27	0.27
D2 (a:f)	Plants from dispersal unit a morph	Proportion of dispersal unit a morph	1.00		
		Proportion of dispersal unit c morph	0.46	1.00	
		Proportion of dispersal unit f morph	-0.47	-1.00**	1.00
		Mass of total plant	0.54*	0.45	-0.45
	Plants from dispersal unit f morph	Proportion of dispersal unit a morph	1.00		
		Proportion of dispersal unit c morph	-0.02	1.00	
		Proportion of dispersal unit f morph	0.03	-0.87**	1.00
		Mass of total plant	-0.09	-0.42	0.35
D2 (c:f)	Plants from dispersal unit c morph	Proportion of dispersal unit a morph	1.00		
		Proportion of dispersal unit c morph	0.89**	1.00	
		Proportion of dispersal unit f morph	-0.90**	-1.00**	1.00
		Mass of total plant	-0.34	-0.39	0.39
	Plants from dispersal unit f morph	Proportion of dispersal unit a morph	1.00		
		Proportion of dispersal unit c morph	0.32	1.00	
		Proportion of dispersal unit f morph	-0.38	-1.00**	1.00
		Mass of total plant	-0.28	-0.34	0.35

Table S5: Pearson correlations between proportion of any two of the three dispersal unit morphs and between mass of total plant and proportion of each dispersal unit morph for *Ceratocarpus arenarius* plants derived from the three dispersal unit morphs in different levels of mixed density (D3). N = 30 for each correlation of plants from each dispersal unit morph. ** P < 0.01.

Plant origin	Characters	Proportion of dispersal unit a morph	Proportion of dispersal unit c morph	Proportion of dispersal unit f morph
Plants from dispersal unit a morph	Proportion of dispersal unit a morph	1.00		
	Proportion of dispersal unit c morph	-0.09	1.00	
	Proportion of dispersal unit f morph	0.04	-1.00**	1.00
	Mass of total plant	-0.27	-0.41	0.42
Plants from dispersal unit c morph	Proportion of dispersal unit a morph	1.00		
	Proportion of dispersal unit c morph	0.13	1.00	
	Proportion of dispersal unit f morph	-0.42	-0.96**	1.00
	Mass of total plant	-0.28	0.23	-0.13
Plants from dispersal unit f morph	Proportion of dispersal unit a morph	1.00		
	Proportion of dispersal unit c morph	-0.15	1.00	
	Proportion of dispersal unit f morph	0.06	-1.00**	1.00
	Mass of total plant	-0.30	-0.36	0.39
