# Supplementary material for: Phenotypic plasticity in diaspore production of a amphi-basicarpic cold desert annual that produces polymorphic diaspores
Source: Sci Rep. 2020 Jul 7;10:11142. doi: 10.1038/s41598-020-67380-0 (PMC7341796; doi:10.1038/s41598-020-67380-0)
Supplement: Supplementary file 3 — Supplementary information [file 41598_2020_67380_MOESM3_ESM.docx]

**Additional information**

**Supplementary information**

**Fig. S1:** Effect of water stress (A), nutrient availability (B) and pure (C) and mixed (D, E) density on mass of dispersal unit morphs a, c and f of *Ceratocarpus arenarius* plants derived from each dispersal unit morph (mean ± 1 s.e.). Only proportion of mass of each dispersal unit morph ≥ 0.05 g and standard errors ≥ 0.04 g are shown. Different uppercase letters indicate significant differences in total mass of the three dispersal unit morphs and different lowercase letters significant differences in mass of each of the three dispersal unit morphs among levels for all treatments of plants from the same dispersal unit morph (P < 0.05). DUa, dispersal unit morph a; DUc, dispersal unit morph c; DUf, dispersal unit morph f; H, high; M, moderate; L, low. (pdf)

**Fig. S2:** Effect of water stress (A), nutrient availability (B) and pure (C) and mixed (D, E) density on proportion of mass of dispersal unit morphs a, c and f of *Ceratocarpus arenarius* plants derived from each dispersal unit morph (mean ± 1 s.e.). Only proportion of mass of each dispersal unit morph ≥ 0.78 % and standard errors ≥ 0.67 % are shown. Different lowercase letters indicate significant differences in multiple range comparison among the levels for all treatments of plants from the same dispersal unit morphs (P < 0.05). DUa, dispersal unit morph a; DUc, dispersal unit morph c; DUf, dispersal unit morph f; H, high; M, moderate; L, low. (pdf)

**Fig. S3:** Effect of water stress (A), nutrient availability (B) and pure (C) and mixed (D, E) density on number of dispersal unit morphs a, c and f with a fruit of *Ceratocarpus arenarius* plants derived from each dispersal unit morph (mean ± 1 s.e.). Only the number of each dispersal unit morph with a fruit ≥ 20.25 and standard errors ≥ 5.66 are shown. Different uppercase letters indicate significant differences in total number of the three dispersal unit morphs with a fruit and different lowercase letters significant differences in multiple range comparison among the levels for all treatments of plants from the same dispersal unit morphs (P < 0.05). DUa, dispersal unit morph a; DUc, dispersal unit morph c; DUf, dispersal unit morph f; H, high; M, moderate; L, low. (pdf)

**Fig. S4:** Effect of water stress (A), nutrient availability (B) and pure (C) and mixed (D, E) density on proportion of dispersal unit morphs a, c and f with a fruit of *Ceratocarpus arenarius* plants derived from each dispersal unit morph (mean ± 1 s.e.). Only the proportion of each dispersal unit morph with a fruit ≥ 0.95 % and standard errors ≥ 0.55 % are shown. Bars with different lowercase letters indicate significant differences in multiple range comparison among the levels for all treatments of plants from the same dispersal unit morphs (P < 0.05). DUa, dispersal unit morph a; DUc, dispersal unit morph c; DUf, dispersal unit morph f; H, high; M, moderate; L, low. (pdf)
